# A New Mechanism of Open System Evolution and Its Entropy Using Unitary Transformations in Noncomposite Qudit Systems

**DOI:** 10.3390/e21080736

**Published:** 2019-07-27

**Authors:** Julio A. López-Saldívar, Octavio Castaños, Margarita A. Man’ko, Vladimir I. Man’ko

**Affiliations:** 1 Instituto de Ciencias Nucleares, Universidad Nacional Autónoma de México, Apdo. Postal 70-543, Ciudad de México 04510, Mexico; 2 Moscow Institute of Physics and Technology, State University, Institutskii Per. 9, Dolgoprudnyi, Moscow 141700, Russia; 3 Lebedev Physical Institute, Leninskii Prospect 53, Moscow 119991, Russia; 4 Department of Physics, Tomsk State University, Lenin Avenue 36, Tomsk 634050, Russia

**Keywords:** entropy, open systems, unitary evolution, qubit, qutrit

## Abstract

The evolution of an open system is usually associated with the interaction of the system with an environment. A new method to study the open-type system evolution of a qubit (two-level atom) state is established. This evolution is determined by a unitary transformation applied to the qutrit (three-level atom) state, which defines the qubit subsystems. This procedure can be used to obtain different qubit quantum channels employing unitary transformations into the qutrit system. In particular, we study the phase damping and spontaneous-emission quantum channels. In addition, we mention a proposal for quasiunitary transforms of qubits, in view of the unitary transform of the total qutrit system. The experimental realization is also addressed. The probability representation of the evolution and its information-entropic characteristics are considered.

## 1. Introduction

The open system evolution of a qudit state is known to be the result of interactions with an environment. Usually, the states of the complete system are thought to evolve by a unitary transformation in the Hilbert space $\hat{H}={\hat{H}}_{q}⊗{\hat{H}}_{env}$, then the density operator of the composite system leads us, using the partial tracing procedure, to the density operator of the subsystem ${\hat{\rho }}_{q}$ (qudit), and its evolution is induced by the unitary evolution of the complete system. In this picture, the qubit state dynamics needs the structure of the Hilbert space $\hat{H}$ corresponding to the presence of two subsystems, qudit and environment [1]. In this work, we suggest a new mechanism to study the open system evolution, which does not demand the complete system to have a subsystem.

We show that for any system without subsystems, there exist a unitary evolution, which due to hidden correlations in the system, evolves according to the Gorini–Kossakowski–Sudarshan–Lindblad equation [2,3,4,5]. We demonstrate this picture using the example of a qutrit (complete system without subsystems), where the open-like evolution is available for their associated qubits.

In previous works [6,7,8,9,10], a new method to define different qubit density matrices from a qudit system was established. This procedure uses the occupation probabilities and transition probability amplitudes for different levels of a qudit system and groups them as if there exists two levels only. This is done by mapping the qudit density matrix to the closest higher even-dimensional density matrix. The partial trace operation then is enacted on the resulting matrix in order to obtain well-defined qubit density matrices.

The obtained qubits have been used to define a new geometric representation of the *d*-dimensional qudit states through *d* Bloch vectors [10] associated with the generated qubits. Furthermore, it has been possible to describe quantum phenomena as the entanglement on a two-qubit system in terms of standard probabilities [9].

The evolution of a qutrit density matrix can provide the quantum channel, which maps the initial state ${\hat{\rho }}_{a}$ onto the density matrix ${\hat{\rho }}_{a}^{\prime }$. The proposed open-type evolution establishes a new mechanism, which will need a special state preparation and a specific unitary operation for the qutrit system, as we will show later on. The experimental possibilities by which one can realize this new mechanism are related to superconducting circuit devices [11,12].

Most quantum computing processes consider a set of pure qubit states, which are transformed by unitary operators, also called gates, that are used to implement different computing algorithms. In this article, instead, we have density matrices (which might be describing a mixed state) of larger qudit systems. The definition of a set of qubit states from a qudit system is similar to the ideas established in [13], where the emulation of a spin system was obtained from qudit states, and in [14], where the quantum logic of qubits was simplified by the use of a higher dimensional Hilbert space; and in general, with all the procedures that make use of larger Hilbert spaces. In this work, we demonstrate that subsystems of qubits defined by larger systems can be used in quantum information. A principal foundation of quantum computation is the study of quantum channels. These channels are linked to unitary transformations of the qubit density matrix. There exist several channels that can describe the interaction between a quantum system and its environment such as the bit-flip, depolarization, spontaneous emission, phase, and amplitude damping channels. For this, the study of quantum channels has been of relevance in the error correction theory of quantum computation [15,16].

Here, we present different examples of quantum channels, which act on the associated qubits to qudit states. These quantum channels have the advantage of being represented as unitary transformations acting in the qudit system, providing the possibility to study the qubits as if they were interacting with an environment.

On the other hand, the study of the interaction of three-level systems with electromagnetic fields has led to the discovery of important phenomena, such as the presence of dark states [17] together with black resonances [18] and electromagnetically-induced transparency [19,20,21]. This is important to our objectives as in some cases, the herein proposed qubit quantum channels can be obtained by a unitary transformation of dark states, suggesting the possibility of checking our results experimentally.

The work is organized as follows: In Section 2, a review of the qubit density matrices that are associated with a qutrit state is given. Furthermore, the association of a unitary transform of the qutrit to the nonunitary transformations of the qubits is studied. In Section 3, the definitions of the qubit phase damping and spontaneous-emission quantum channels are reviewed. Later, the unitary transformations of a qutrit system are explicitly given, which yields the phase damping and spontaneous-emission channels on the associated qubits. A way to obtain a quasi-unitary transformation on the qubits is also explored. The change of entropy associated with the nonunitary evolution of the qubits is discussed in Section 4. Finally, some concluding remarks are given.

## 2. Nonunitary Evolution for the Qubit Decomposition of Qutrit States

In a previous work [10], we showed the existence of six different qubit states associated with a general qutrit density matrix:

$$\hat{\rho }=\left(\begin{array}{ccc} \rho_{11} & \rho_{12} & \rho_{13}\\ \rho_{21} & \rho_{22} & \rho_{23}\\ \rho_{31} & \rho_{32} & \rho_{33} \end{array}\right)\;.$$

To define these states, different maps of $\hat{\rho }$ to a 4 × 4 density matrix, with one row and one column equal to zero (in such a way that ensures an eigenvalue equal to zero), were used. Then, the partial trace of the resulting 4 × 4 matrix was performed as if it was describing a two-qubit system. The obtained qubit partial density operators can be explicitly written as:

(1) $$\begin{array}{c} {\hat{\rho }}_{1}=\left(\begin{array}{cc} 1-\rho_{33} & \rho_{13}\\ \rho_{31} & \rho_{33} \end{array}\right),\;{\hat{\rho }}_{2}=\left(\begin{array}{cc} 1-\rho_{22} & \rho_{12}\\ \rho_{21} & \rho_{22} \end{array}\right),\;{\hat{\rho }}_{3}=\left(\begin{array}{cc} \rho_{11} & \rho_{13}\\ \rho_{31} & 1-\rho_{11} \end{array}\right),\\ {\hat{\rho }}_{4}=\left(\begin{array}{cc} \rho_{22} & \rho_{23}\\ \rho_{32} & 1-\rho_{22} \end{array}\right),\;{\hat{\rho }}_{5}=\left(\begin{array}{cc} \rho_{11} & \rho_{12}\\ \rho_{21} & 1-\rho_{11} \end{array}\right),\;{\hat{\rho }}_{6}=\left(\begin{array}{cc} 1-\rho_{33} & \rho_{23}\\ \rho_{32} & \rho_{33} \end{array}\right). \end{array}$$

The qubit states can be characterized in different sets by their corresponding von Neumann entropy $S_{k}=-Tr\;\rho_{k}\ln\rho_{k}$, with k=1,2,…,6. These qubits correspond to the reduction of the three-level system to different two-level systems by the summation of the population probabilities of two levels into one.

When the qutrit state is transformed using a general three-dimensional unitary matrix $\hat{U}$, i.e., ${\hat{\rho }}^{\prime }={\hat{U}}^{†}\;\hat{\rho }\;\hat{U}$, the qubits in Equation (1) are transformed in a nonunitary way. The transformed qubit density matrices can be written by the following expressions:

(2) $$\begin{array}{ccc} {\hat{\rho }}_{1}^{\prime } & = & \frac{1}{D}\left(\begin{array}{cc} D-M_{3,1}N_{1,3}+M_{2,1}N_{2,3}-M_{1,1}N_{3,3} & M_{3,3}N_{1,3}-M_{2,3}N_{2,3}+M_{1,3}N_{3,3}\\ M_{3,1}N_{1,1}-M_{2,1}N_{2,1}+M_{1,1}N_{3,1} & M_{3,1}N_{1,3}-M_{2,1}N_{2,3}+M_{1,1}N_{3,3} \end{array}\right),\\ {\hat{\rho }}_{2}^{\prime } & = & \frac{1}{D}\left(\begin{array}{cc} D+M_{3,2}N_{1,2}-M_{2,2}N_{2,2}+M_{1,2}N_{3,2} & M_{3,3}N_{1,2}-M_{2,3}N_{2,2}+M_{1,3}N_{3,2}\\ -M_{3,2}N_{1,1}+M_{2,2}N_{2,1}-M_{1,2}N_{3,1} & -M_{3,2}N_{1,2}+M_{2,2}N_{2,2}-M_{1,2}N_{3,2} \end{array}\right),\\ {\hat{\rho }}_{3}^{\prime } & = & \frac{1}{D}\left(\begin{array}{cc} M_{3,3}N_{1,1}-M_{2,3}N_{2,1}+M_{1,3}N_{3,1} & M_{3,3}N_{1,3}-M_{2,3}N_{2,3}+M_{1,3}N_{3,3}\\ M_{3,1}N_{1,1}-M_{2,1}N_{2,1}+M_{1,1}N_{3,1} & D-M_{3,3}N_{1,1}+M_{2,3}N_{2,1}-M_{1,3}N_{3,1} \end{array}\right),\\ {\hat{\rho }}_{4}^{\prime } & = & \frac{1}{D}\left(\begin{array}{cc} -M_{3,2}N_{1,2}+M_{2,2}N_{2,2}-M_{1,2}N_{3,2} & -M_{3,2}N_{1,3}+M_{2,2}N_{2,3}-M_{1,2}N_{3,3}\\ M_{3,1}N_{1,2}-M_{2,1}N_{2,2}+M_{1,1}N_{3,2} & D+M_{3,2}N_{1,2}-M_{2,2}N_{2,2}+M_{1,2}N_{3,2} \end{array}\right),\\ {\hat{\rho }}_{5}^{\prime } & = & \frac{1}{D}\left(\begin{array}{cc} M_{3,3}N_{1,1}-M_{2,3}N_{2,1}+M_{1,3}N_{3,1} & M_{3,3}N_{1,2}-M_{2,3}N_{2,2}+M_{1,3}N_{3,2}\\ -M_{3,2}N_{1,1}+M_{2,2}N_{2,1}-M_{1,2}N_{3,1} & D-M_{3,3}N_{1,1}+M_{2,3}N_{2,1}-M_{1,3}N_{3,1} \end{array}\right),\\ {\hat{\rho }}_{6}^{\prime } & = & \frac{1}{D}\left(\begin{array}{cc} D-M_{3,1}N_{1,3}+M_{2,1}N_{2,3}-M_{1,1}N_{3,3} & -M_{3,2}N_{1,3}+M_{2,2}N_{2,3}-M_{1,2}N_{3,3}\\ M_{3,1}N_{1,2}-M_{2,1}N_{2,2}+M_{1,1}N_{3,2} & M_{3,1}N_{1,3}-M_{2,1}N_{2,3}+M_{1,1}N_{3,3} \end{array}\right), \end{array}$$

where $N_{jk}={\left(\hat{\rho }\hat{U}\right)}_{jk}$, *D* is the determinant of $\hat{U}$, and $M_{jk}$ are the components of the minors of matrix $\hat{U}$, i.e., its elements are the determinants after eliminating the ${\left(4-j\right)}^{\mathrm{th}}$ row and ${\left(4-k\right)}^{\mathrm{th}}$ column of $\hat{U}$. The transformed states are characterized into different sets by their corresponding transformed entropies $S_{k}^{\prime }=-Tr\;\rho_{k}^{\prime }\ln\rho_{k}^{\prime }$. We emphasize that the resulting qubit density matrices are associated, in general, with a nonunitary evolution of the original qubits. This fact establishes a new mechanism to obtain the open-like system evolution in a noncomposite qutrit system. Additionally, this procedure can be extended to any qudit system, in view of the general definition of the qubit density matrices obtained from a qudit system [10].

In [9], we discussed that a two-qubit density matrix with one of its rows and columns equal to zero describes separable states, if one of the off-diagonal terms is equal to zero, for example, the state:

$$\hat{\rho }=\left(\begin{array}{cccc} \rho_{11} & \rho_{12} & \rho_{13} & 0\\ \rho_{21} & \rho_{22} & \rho_{23} & 0\\ \rho_{31} & \rho_{32} & \rho_{33} & 0\\ 0 & 0 & 0 & 0 \end{array}\right)$$

is separable iff $\rho_{23}=0$. To show this, one can consider the previous density matrix to be in the standard two-qubit representation |00〉, |01〉, |10〉, and |11〉. It can be seen that the partial transpose operation [22] implies the change $\rho_{12}↔\rho_{21}$, and for this reason, the eigenvalues of $\hat{\rho }$ with $\rho_{23}=0$ are equal to the eigenvalues of its partial transpose. As the partial transpose is a nonnegative operator, then the system is separable. The separability implies the invariance of the partial density matrices under local unitary transformations. As this two-qubit density matrix has a pair of row-column with a diagonal term equal to zero, the correspondence with a qutrit density matrix can be made. On the other hand, the correspondence between two-qubit local unitary transformations and qutrit unitary transformations can be made in the same way, e.g., by eliminating one row and one column of the two-qubit local transformation. This procedure allows us to define different unitary transformations that almost leave the qubits in Expression (1) invariant.

## 3. Phase Damping and Spontaneous-Emission Channels

It is known that the interaction of a qubit system with an environment leads to several physical phenomena such as dissipation and decoherence in the qubit subsystem; an example of these interactions is the phase damping channel. In this channel, the evolution of the qubit plus environment (${|\cdots \rangle }_{q}{|\cdots \rangle }_{e}$) is given by a unitary transformation $\hat{T}$, which acts differently if the qubit is in the ground or excited state, according to the following rules: $\hat{T}{(|0\rangle }_{q}{|0\rangle }_{e})=\sqrt{1-p}{|0\rangle }_{q}{|0\rangle }_{e}+\sqrt{p}{|0\rangle }_{q}{|1\rangle }_{e}$ and $\hat{T}{(|1\rangle }_{q}{|0\rangle }_{e})=\sqrt{1-p}{|0\rangle }_{q}{|0\rangle }_{e}+\sqrt{p}{|0\rangle }_{q}{|2\rangle }_{e}$ with *p* being a probability, i.e., the environment subsystem goes to a superposition of the states (${|0\rangle }_{e}$, ${|1\rangle }_{e}$), or to (${|0\rangle }_{e}$, ${|2\rangle }_{e}$), if the environment is in ${|0\rangle }_{e}$, or ${|1\rangle }_{e}$, respectively [15,23]. This two-qubit unitary transformations result in a nonunitary change when the partial trace over the environment subsystem is taken:

$$\left(\begin{array}{cc} 1-\rho_{22} & \rho_{12}\\ \rho_{12}^{*} & \rho_{22} \end{array}\right)\to \left(\begin{array}{cc} 1-\rho_{22} & \rho_{12}\left(1-p\right)\\ \rho_{12}^{*}\left(1-p\right) & \rho_{22} \end{array}\right)\;.$$

When the map is applied a very large number of times (→∞), it is straightforward that the initial state tends to the completely decoherent state:

$$\left(\begin{array}{cc} 1-\rho_{22} & \rho_{12}\\ \rho_{12}^{*} & \rho_{22} \end{array}\right)\to \left(\begin{array}{cc} 1-\rho_{22} & 0\\ 0 & \rho_{22} \end{array}\right)\;,$$

with an exponential convergence.

The other example is the spontaneous-emission (also called the amplitude-damping) quantum channel. In this channel, the dynamics of the qubit system plus the environment is determined by a unitary transform $\hat{T}$, which only acts if the qubit system is in the excited state ${|1\rangle }_{q}$, according to the following rules: $\hat{T}{(|0\rangle }_{q}{|0\rangle }_{e}{)=|0\rangle }_{q}{|0\rangle }_{e}$ and $\hat{T}{(|1\rangle }_{q}{|0\rangle }_{e})=\sqrt{1-p}{|1\rangle }_{q}{|0\rangle }_{e}+\sqrt{p}{|0\rangle }_{q}{|1\rangle }_{e}$, where *p* is the probability [15,23]. This channel then defines a nonunitary evolution over the qubit subsystem, which transforms the qubit density matrix as follows:

$$\left(\begin{array}{cc} 1-\rho_{22} & \rho_{12}\\ \rho_{12}^{*} & \rho_{22} \end{array}\right)\to \left(\begin{array}{cc} 1-\left(1-p\right)\rho_{22} & \rho_{12}\sqrt{1-p}\\ \rho_{12}^{*}\sqrt{1-p} & \left(1-p\right)\rho_{22} \end{array}\right)\;.$$

If this channel is applied a very large number of times (→∞), the density matrix converges to a ground state, i.e.,

$$\left(\begin{array}{cc} 1-\rho_{22} & \rho_{12}\\ \rho_{12}^{*} & \rho_{22} \end{array}\right)\to \left(\begin{array}{cc} 1 & 0\\ 0 & 0 \end{array}\right)\;.$$

In addition to these examples, there exists another type of quantum channel defined in the theory of interaction between a quantum system and an environment, which can be considered [15,23].

It is possible to demonstrate that phase damping and spontaneous-emission quantum channels for qubits ${\hat{\rho }}_{1},\ldots ,{\hat{\rho }}_{6}$ in Equation (1) can be obtained by the use of particular unitary transformations of a qutrit state $\hat{\rho }$. To justify this, we assumed a two-qubit quantum system where one of the levels cannot be populated, i.e., the 4 × 4 density matrix has an eigenvalue equal to zero, e.g.,

(3) $$\hat{\rho }=\left(\begin{array}{cccc} \rho_{11} & \rho_{12} & \rho_{13} & 0\\ \rho_{21} & \rho_{22} & 0 & 0\\ \rho_{31} & 0 & \rho_{33} & 0\\ 0 & 0 & 0 & 0 \end{array}\right);$$

it is clear that this density matrix is separable since $\rho_{23}=\rho_{32}^{*}=0$. The partial density matrices can be operated locally by unitary transformations of the form ${\hat{u}}_{1}⊗{\hat{u}}_{2}$. When only one of the qubits is operated, i.e., when the unitary matrix corresponds to a controlled operation [15]: ${\hat{u}}_{1}=\hat{I}$ or ${\hat{u}}_{2}=\hat{I}$. If ${\hat{u}}_{2}=\hat{I}$, then the unitary transformation only operates over the second qubit,

(4) $$\hat{u}=\left(\begin{array}{cccc} u_{11} & u_{12} & 0 & 0\\ u_{21} & u_{22} & 0 & 0\\ 0 & 0 & u_{11} & u_{12}\\ 0 & 0 & u_{21} & u_{22} \end{array}\right).$$

By means of this type of unitary matrix, one can define an operation in the qutrit system that approximately only affects ${\hat{\rho }}_{2}$. This is done by ignoring the fourth row and the fourth column of (3); the resulting qutrit state is then operated by the unitary matrix resulting from the elimination of the fourth row and the fourth column of Equation (4). For the operator to be still unitary, the (3,3) entry must be replaced by one. Following these and other analogous arguments, we study the application of the unitary transforms:

(5) $${\hat{U}}_{1}=\left(\begin{array}{ccc} u_{11} & u_{12} & 0\\ u_{21} & u_{22} & 0\\ 0 & 0 & 1 \end{array}\right),\;{\hat{U}}_{2}=\left(\begin{array}{ccc} u_{11} & 0 & u_{12}\\ 0 & 1 & 0\\ u_{21} & 0 & u_{22} \end{array}\right),\;{\hat{U}}_{3}=\left(\begin{array}{ccc} 1 & 0 & 0\\ 0 & u_{11} & u_{12}\\ 0 & u_{21} & u_{22} \end{array}\right)\;$$

on the qutrit density matrices:

(6) $${\hat{\sigma }}_{1}=\left(\begin{array}{ccc} \rho_{11} & \rho_{12} & \rho_{13}\\ \rho_{21} & \rho_{22} & 0\\ \rho_{31} & 0 & \rho_{33} \end{array}\right),\;{\hat{\sigma }}_{2}=\left(\begin{array}{ccc} \rho_{11} & 0 & \rho_{13}\\ 0 & \rho_{22} & \rho_{23}\\ \rho_{31} & \rho_{32} & \rho_{33} \end{array}\right),\;{\hat{\sigma }}_{3}=\left(\begin{array}{ccc} \rho_{11} & \rho_{12} & 0\\ \rho_{21} & \rho_{22} & \rho_{23}\\ 0 & \rho_{23} & \rho_{33} \end{array}\right)\;.$$

The unitary transformations in Equation (5) can be enacted on any of the density matrices in Equation (6), which define a nonunitary transformation of the qubits defined in Equation (1). These qubit transformations are found by the substitution of Equations (5) and (6) into Equation (2), e.g., the unitary transformation ${\hat{U}}_{1}^{†}{\hat{\sigma }}_{1}{\hat{U}}_{1}$ results in the following transformations of the qubits:

$$\begin{array}{c} {\hat{\rho }}_{1}^{\prime }=\left(\begin{array}{cc} 1-\rho_{33} & \rho_{13}\;u_{11}^{*}\\ \rho_{31}\;u_{11} & \rho_{33} \end{array}\right),\\ {\hat{\rho }}_{2}^{\prime }=\left(\begin{array}{cc} 1-u_{12}^{*}{\left({\hat{\sigma }}_{1}{\hat{U}}_{1}\right)}_{12}-u_{22}^{*}{\left({\hat{\sigma }}_{1}{\hat{U}}_{1}\right)}_{22} & u_{11}^{*}{\left({\hat{\sigma }}_{1}{\hat{U}}_{1}\right)}_{12}+u_{21}^{*}{\left({\hat{\sigma }}_{1}{\hat{U}}_{1}\right)}_{22}\\ u_{12}^{*}{\left({\hat{\sigma }}_{1}{\hat{U}}_{1}\right)}_{11}+u_{22}^{*}{\left({\hat{\sigma }}_{1}{\hat{U}}_{1}\right)}_{21} & u_{12}^{*}{\left({\hat{\sigma }}_{1}{\hat{U}}_{1}\right)}_{12}+u_{22}^{*}{\left({\hat{\sigma }}_{1}{\hat{U}}_{1}\right)}_{22} \end{array}\right),\\ {\hat{\rho }}_{3}^{\prime }=\left(\begin{array}{cc} u_{11}^{*}{\left({\hat{\sigma }}_{1}{\hat{U}}_{1}\right)}_{11}+u_{21}^{*}{\left({\hat{\sigma }}_{1}{\hat{U}}_{1}\right)}_{21} & \rho_{13}\;u_{11}^{*}\\ \rho_{31}\;u_{11} & 1-u_{11}^{*}{\left({\hat{\sigma }}_{1}{\hat{U}}_{1}\right)}_{11}-u_{21}^{*}{\left({\hat{\sigma }}_{1}{\hat{U}}_{1}\right)}_{21} \end{array}\right),\\ {\hat{\rho }}_{4}^{\prime }=\left(\begin{array}{cc} u_{12}^{*}{\left({\hat{\sigma }}_{1}{\hat{U}}_{1}\right)}_{12}+u_{22}^{*}{\left({\hat{\sigma }}_{1}{\hat{U}}_{1}\right)}_{22} & \rho_{13}u_{12}^{*}\\ \rho_{31}u_{12} & 1-u_{12}^{*}{\left({\hat{\sigma }}_{1}{\hat{U}}_{1}\right)}_{12}-u_{22}^{*}{\left({\hat{\sigma }}_{1}{\hat{U}}_{1}\right)}_{22} \end{array}\right)\;,\\ {\hat{\rho }}_{5}^{\prime }=\left(\begin{array}{cc} u_{11}^{*}{\left({\hat{\sigma }}_{1}{\hat{U}}_{1}\right)}_{11}+u_{21}^{*}{\left({\hat{\sigma }}_{1}{\hat{U}}_{1}\right)}_{21} & u_{11}^{*}{\left({\hat{\sigma }}_{1}{\hat{U}}_{1}\right)}_{12}+u_{21}^{*}{\left({\hat{\sigma }}_{1}{\hat{U}}_{1}\right)}_{22}\\ u_{12}^{*}{\left({\hat{\sigma }}_{1}{\hat{U}}_{1}\right)}_{11}\left(r11u11+r12u21\right)+u_{22}^{*}{\left({\hat{\sigma }}_{1}{\hat{U}}_{1}\right)}_{21} & 1-u_{11}^{*}{\left({\hat{\sigma }}_{1}{\hat{U}}_{1}\right)}_{11}-u_{21}^{*}{\left({\hat{\sigma }}_{1}{\hat{U}}_{1}\right)}_{21} \end{array}\right),\\ {\hat{\rho }}_{6}^{\prime }=\left(\begin{array}{cc} 1-\rho_{33} & \rho_{13}\;u_{12}^{*}\\ \rho_{31}\;u_{12} & \rho_{33} \end{array}\right). \end{array}$$

From these results, one can notice that the transformed qubits ${\hat{\rho }}_{1}^{\prime }$ and ${\hat{\rho }}_{6}^{\prime }$ correspond to the phase damping channel of ${\hat{\rho }}_{1}$ with different damping parameters. Furthermore, the qubit states ${\hat{\rho }}_{2}^{\prime }$, ${\hat{\rho }}_{5}^{\prime }$ can be seen as quasi-unitary transformations of the initial states ${\hat{\rho }}_{2}$, ${\hat{\rho }}_{5}$, respectively. In a similar way, one can obtain all the possible unitary transformations of the density matrices in Equation (6). These transformations lead to the identification of two types of quantum channels: the phase damping and a quasi-unitary operation described below.

The unitary transformation over the density matrices ${\hat{\sigma }}_{1}$, ${\hat{\sigma }}_{2}$, and ${\hat{\sigma }}_{3}$ results in a change over their associated qubits ${\hat{\rho }}_{1},\ldots ,{\hat{\rho }}_{6}$, to ${\hat{\rho }}_{1}^{\prime },\ldots ,{\hat{\rho }}_{6}^{\prime }$, which denote the qubits after the transformation. We have found the following interesting expressions:

(7) $$\begin{array}{c} {\hat{U}}_{1}^{†}\hat{\sigma_{1}}{\hat{U}}_{1}\Rightarrow {\hat{\rho }}_{1}^{\prime }=\left(\begin{array}{cc} 1-\rho_{33} & u_{11}^{*}\rho_{13}\\ u_{11}\rho_{31} & \rho_{33} \end{array}\right),\;{\hat{\rho }}_{6}^{\prime }=\left(\begin{array}{cc} 1-\rho_{33} & u_{12}^{*}\rho_{13}\\ u_{12}\rho_{31} & \rho_{33} \end{array}\right);\\ {\hat{U}}_{2}^{†}\hat{\sigma_{1}}{\hat{U}}_{2}\Rightarrow {\hat{\rho }}_{2}^{\prime }=\left(\begin{array}{cc} 1-\rho_{22} & u_{11}^{*}\rho_{12}\\ u_{11}\rho_{21} & \rho_{22} \end{array}\right),\;{\hat{\rho }}_{4}^{\prime }=\left(\begin{array}{cc} 1-\rho_{33} & u_{12}^{*}\rho_{12}\\ u_{12}\rho_{21} & \rho_{33} \end{array}\right);\\ {\hat{U}}_{2}^{†}\hat{\sigma_{2}}{\hat{U}}_{2}\Rightarrow {\hat{\rho }}_{2}^{\prime }=\left(\begin{array}{cc} 1-\rho_{22} & u_{21}^{*}\rho_{32}\\ u_{21}\rho_{23} & \rho_{22} \end{array}\right),\;{\hat{\rho }}_{4}^{\prime }=\left(\begin{array}{cc} \rho_{22} & u_{22}\rho_{23}\\ u_{22}^{*}\rho_{32} & 1-\rho_{22} \end{array}\right);\\ {\hat{U}}_{3}^{†}\hat{\sigma_{2}}{\hat{U}}_{3}\Rightarrow {\hat{\rho }}_{3}^{\prime }=\left(\begin{array}{cc} \rho_{11} & u_{22}\rho_{13}\\ u_{22}^{*}\rho_{31} & 1-\rho_{11} \end{array}\right),\;{\hat{\rho }}_{5}^{\prime }=\left(\begin{array}{cc} \rho_{11} & u_{21}\rho_{13}\\ u_{21}^{*}\rho_{31} & 1-\rho_{11} \end{array}\right);\\ {\hat{U}}_{1}^{†}\hat{\sigma_{3}}{\hat{U}}_{1}\Rightarrow {\hat{\rho }}_{1}^{\prime }=\left(\begin{array}{cc} 1-\rho_{33} & u_{21}^{*}\rho_{23}\\ u_{21}\rho_{32} & \rho_{33} \end{array}\right),\;{\hat{\rho }}_{6}^{\prime }=\left(\begin{array}{cc} 1-\rho_{33} & u_{22}^{*}\rho_{23}\\ u_{22}\rho_{32} & \rho_{33} \end{array}\right);\\ {\hat{U}}_{3}^{†}\hat{\sigma_{3}}{\hat{U}}_{3}\Rightarrow {\hat{\rho }}_{3}^{\prime }=\left(\begin{array}{cc} \rho_{11} & u_{12}\rho_{12}\\ u_{12}^{*}\rho_{21} & 1-\rho_{11} \end{array}\right),\;{\hat{\rho }}_{5}^{\prime }=\left(\begin{array}{cc} \rho_{11} & u_{11}\rho_{12}\\ u_{11}^{*}\rho_{21} & 1-\rho_{11} \end{array}\right). \end{array}$$

In most of the cases, the resulting qubits ${\hat{\rho }}_{j}^{\prime }$ correspond to the phase damping quantum channel of ${\hat{\rho }}_{j}$, as can be seen in Expression (8). In this channel, the probability amplitudes given by the original off-diagonal terms of the qubits are multiplied by a number. The damping parameters are associated with different entries of the unitary transformation $u_{jk}$, which in general are complex numbers. When the unitary transformation correspond to a real matrix, then the expression for the standard phase damping map is obtained. As you can see in Equation (8), in some cases, the unitary transformations leads to the quantum channel of another qubit, e.g., after the application of ${\hat{U}}_{1}$ to ${\hat{\sigma }}_{1}$, the qubit ${\hat{\rho }}_{6}^{\prime }$ is the phase damping channel of ${\hat{\rho }}_{1}$. Furthermore, in some other cases, the obtained density matrices correspond to transformations similar to the phase damping channel of matrices outside the ones in Equation (1), e.g., ${\hat{\rho }}_{4}^{\prime }$ after the application of ${\hat{U}}_{2}$ to ${\hat{\sigma }}_{1}$. Although these matrices seem unrelated, they have the same form as the phase damping channel. In the case of $\hat{U}$ being a rotation matrix with a time-dependent angle θ=ωt, the original qubit states can be recovered at the time t=2πl/ω, l=0,1,2,….

The unitary transformations (${\hat{U}}_{1}$, ${\hat{U}}_{2}$, ${\hat{U}}_{3}$) previously described can also lead to quasi-unitary transformations of the qubits. In particular, for the unitary transformation ${\hat{U}}_{1}^{†}{\hat{\sigma }}_{1}{\hat{U}}_{1}$, one gets the quasi-unitary transformations:

(8) $$\begin{array}{ccc} {\hat{\rho }}_{2}^{\prime } & = & {\hat{U}}^{†}{\hat{\rho }}_{2}\;\hat{U}+\rho_{33}\left(\begin{array}{cc} |u_{12}{|}^{2} & -u_{11}^{*}u_{12}\\ -u_{11}u_{12}^{*} & -|u_{12}{|}^{2} \end{array}\right)\;,\\ {\hat{\rho }}_{5}^{\prime } & = & {\hat{U}}^{†}{\hat{\rho }}_{5}\;\hat{U}+\rho_{33}\left(\begin{array}{cc} -|u_{21}{|}^{2} & -u_{21}^{*}u_{22}\\ -u_{21}u_{22}^{*} & |u_{21}{|}^{2} \end{array}\right)\;, \end{array}$$

with $\hat{U}=\left(\begin{array}{cc} u_{11} & u_{12}\\ u_{21} & u_{22} \end{array}\right)$ being a two-dimensional unitary transformation. For the other qubits, one can also define quasi-unitary transformations as follows:

(a) From the qutrit unitary transformation ${\hat{U}}_{1}^{†}{\hat{\sigma }}_{3}{\hat{U}}_{1}$,
  (9) $$\begin{array}{ccc} {\hat{\rho }}_{2}^{\prime } & = & {\hat{U}}^{†}{\hat{\rho }}_{2}\;\hat{U}+\rho_{33}\left(\begin{array}{cc} -|u_{12}{|}^{2} & u_{11}^{*}u_{12}\\ u_{11}u_{12}^{*} & |u_{12}{|}^{2} \end{array}\right)\;,\\ {\hat{\rho }}_{5}^{\prime } & = & {\hat{U}}^{†}{\hat{\rho }}_{5}\;\hat{U}+\rho_{33}\left(\begin{array}{cc} |u_{21}{|}^{2} & u_{21}^{*}u_{22}\\ u_{21}u_{22}^{*} & -|u_{21}{|}^{2} \end{array}\right)\;, \end{array}$$
(b) For the transformation ${\hat{U}}_{2}^{†}{\hat{\sigma }}_{1}{\hat{U}}_{2}$,
  (10) $$\begin{array}{ccc} {\hat{\rho }}_{1}^{\prime } & = & {\hat{U}}^{†}{\hat{\rho }}_{1}\;\hat{U}+\rho_{22}\left(\begin{array}{cc} |u_{12}{|}^{2} & -u_{11}^{*}u_{12}\\ -u_{11}u_{12}^{*} & -|u_{12}{|}^{2} \end{array}\right)\;,\\ {\hat{\rho }}_{3}^{\prime } & = & {\hat{U}}^{†}{\hat{\rho }}_{3}\;\hat{U}+\rho_{22}\left(\begin{array}{cc} -|u_{21}{|}^{2} & -u_{21}^{*}u_{22}\\ -u_{21}u_{22}^{*} & |u_{21}{|}^{2} \end{array}\right)\;. \end{array}$$
(c) For the transformation ${\hat{U}}_{2}^{†}{\hat{\sigma }}_{2}{\hat{U}}_{2}$,
  (11) $$\begin{array}{ccc} {\hat{\rho }}_{1}^{\prime } & = & {\hat{U}}^{†}{\hat{\rho }}_{1}\;\hat{U}+\rho_{22}\left(\begin{array}{cc} |u_{12}{|}^{2} & -u_{11}^{*}u_{12}\\ -u_{11}u_{12}^{*} & -|u_{12}{|}^{2} \end{array}\right)\;,\\ {\hat{\rho }}_{3}^{\prime } & = & {\hat{U}}^{†}{\hat{\rho }}_{3}\;\hat{U}+\rho_{22}\left(\begin{array}{cc} -|u_{21}{|}^{2} & -u_{21}^{*}u_{22}\\ -u_{21}u_{22}^{*} & |u_{21}{|}^{2} \end{array}\right)\;, \end{array}$$
(d) From ${\hat{U}}_{3}^{†}{\hat{\sigma }}_{2}{\hat{U}}_{3}$,
  (12) $$\begin{array}{ccc} {\hat{\rho }}_{4}^{\prime } & = & {\hat{U}}^{†}{\hat{\rho }}_{4}\;\hat{U}+\rho_{11}\left(\begin{array}{cc} -|u_{21}{|}^{2} & -u_{12}^{*}u_{22}\\ -u_{21}u_{22}^{*} & |u_{21}{|}^{2} \end{array}\right)\;,\\ {\hat{\rho }}_{6}^{\prime } & = & {\hat{U}}^{†}{\hat{\rho }}_{6}\;\hat{U}+\rho_{11}\left(\begin{array}{cc} -|u_{12}{|}^{2} & u_{11}^{*}u_{12}\\ u_{11}u_{12}^{*} & |u_{12}{|}^{2} \end{array}\right)\;. \end{array}$$
(e) Finally, for ${\hat{U}}_{3}^{†}{\hat{\sigma }}_{3}{\hat{U}}_{3}$,
  (13) $$\begin{array}{ccc} {\hat{\rho }}_{4}^{\prime } & = & {\hat{U}}^{†}{\hat{\rho }}_{4}\;\hat{U}+\rho_{11}\left(\begin{array}{cc} -|u_{21}{|}^{2} & -u_{12}^{*}u_{22}\\ -u_{21}u_{22}^{*} & |u_{21}{|}^{2} \end{array}\right)\;,\\ {\hat{\rho }}_{6}^{\prime } & = & {\hat{U}}^{†}{\hat{\rho }}_{6}\;\hat{U}+\rho_{11}\left(\begin{array}{cc} -|u_{12}{|}^{2} & u_{11}^{*}u_{12}\\ u_{11}u_{12}^{*} & |u_{12}{|}^{2} \end{array}\right)\;, \end{array}$$

For all the cases, $\hat{U}$ is a two-dimensional unitary transformation.

As in the phase-damping case, one can think of a rotation matrix with a time-dependent angle θ=ωt as the unitary operation, i.e.,

$$\hat{U}=\left(\begin{array}{cc} \cos(\omega t) & -\sin(\omega t)\\ \sin(\omega t) & \cos(\omega t) \end{array}\right)\;,$$

which, in the case where t≈0, results in the following transformations:

(14) $${\hat{\rho }}_{j}^{\prime }={\hat{U}}^{†}{\hat{\rho }}_{j}\;\hat{U}-\rho_{kk}\;\omega \;t\;{\hat{\sigma }}_{x}+O\left(t^{2}\right)\;,$$

where ${\hat{\sigma }}_{x}$ is the Pauli matrix and $\rho_{kk}$ is a diagonal component of $\hat{\rho }$, which depends on *j*. Its value is k=2 for j=1,3, k=3 for j=2,5, and k=1 for j=4,6. It is necessary to point out that, for ${\hat{\rho }}_{5}^{\prime }$ associated with ${\hat{U}}_{1}^{†}{\hat{\sigma }}_{3}{\hat{U}}_{1}$, we need to replace $\rho_{33}$ with $-\rho_{33}$ in Equation (14).

In the case where the density matrices correspond to states, where one of the accessible levels is not occupied, i.e.,

(15) $${\hat{\sigma }}_{4}=\left(\begin{array}{ccc} \rho_{11} & \rho_{12} & 0\\ \rho_{21} & \rho_{22} & 0\\ 0 & 0 & 0 \end{array}\right),\;{\hat{\sigma }}_{5}=\left(\begin{array}{ccc} \rho_{11} & 0 & \rho_{13}\\ 0 & 0 & 0\\ \rho_{31} & 0 & \rho_{33} \end{array}\right),\;{\hat{\sigma }}_{6}=\left(\begin{array}{ccc} 0 & 0 & 0\\ 0 & \rho_{22} & \rho_{23}\\ 0 & \rho_{32} & \rho_{33} \end{array}\right),$$

we obtain the expressions:

(16) $$\begin{array}{c} {\hat{U}}_{2}^{†}{\hat{\sigma }}_{4}{\hat{U}}_{2}\Rightarrow {\hat{\rho }}_{5}^{\prime }=\left(\begin{array}{cc} \rho_{11}{\left|u_{11}\right|}^{2} & \rho_{12}u_{11}^{*}\\ \rho_{21}u_{11} & 1-\rho_{11}{\left|u_{11}\right|}^{2} \end{array}\right),\;{\hat{\rho }}_{6}^{\prime }=\left(\begin{array}{cc} 1-\rho_{11}{\left|u_{12}\right|}^{2} & \rho_{21}u_{12}\\ \rho_{12}u_{12}^{*} & \rho_{11}{\left|u_{12}\right|}^{2} \end{array}\right),\\ {\hat{U}}_{3}^{†}{\hat{\sigma }}_{4}{\hat{U}}_{3}\Rightarrow {\hat{\rho }}_{1}^{\prime }=\left(\begin{array}{cc} 1-\rho_{22}{\left|u_{12}\right|}^{2} & \rho_{12}u_{12}\\ \rho_{21}u_{12}^{*} & \rho_{22}{\left|u_{12}\right|}^{2} \end{array}\right),\;{\hat{\rho }}_{2}^{\prime }=\left(\begin{array}{cc} 1-\rho_{22}{\left|u_{11}\right|}^{2} & \rho_{12}u_{11}\\ \rho_{21}u_{11}^{*} & \rho_{22}{\left|u_{11}\right|}^{2} \end{array}\right),\\ {\hat{U}}_{1}^{†}{\hat{\sigma }}_{5}{\hat{U}}_{1}\Rightarrow {\hat{\rho }}_{3}^{\prime }=\left(\begin{array}{cc} \rho_{11}{\left|u_{11}\right|}^{2} & \rho_{13}u_{11}^{*}\\ \rho_{31}u_{11} & 1-\rho_{11}{\left|u_{11}\right|}^{2} \end{array}\right),\;{\hat{\rho }}_{4}^{\prime }=\left(\begin{array}{cc} \rho_{11}{\left|u_{12}\right|}^{2} & \rho_{13}u_{12}^{*}\\ \rho_{31}u_{12} & 1-\rho_{11}{\left|u_{12}\right|}^{2} \end{array}\right),\\ {\hat{U}}_{3}^{†}{\hat{\sigma }}_{5}{\hat{U}}_{3}\Rightarrow {\hat{\rho }}_{1}^{\prime }=\left(\begin{array}{cc} 1-\rho_{33}{\left|u_{22}\right|}^{2} & \rho_{13}u_{22}\\ \rho_{31}u_{22}^{*} & \rho_{33}{\left|u_{22}\right|}^{2} \end{array}\right),\;{\hat{\rho }}_{2}^{\prime }=\left(\begin{array}{cc} 1-\rho_{33}{\left|u_{21}\right|}^{2} & \rho_{13}u_{21}\\ \rho_{31}u_{21}^{*} & \rho_{33}{\left|u_{21}\right|}^{2} \end{array}\right),\\ {\hat{U}}_{1}^{†}{\hat{\sigma }}_{6}{\hat{U}}_{1}\Rightarrow {\hat{\rho }}_{3}^{\prime }=\left(\begin{array}{cc} \rho_{22}{\left|u_{21}\right|}^{2} & \rho_{23}u_{21}^{*}\\ \rho_{32}u_{21} & 1-\rho_{22}{\left|u_{21}\right|}^{2} \end{array}\right),\;{\hat{\rho }}_{4}^{\prime }=\left(\begin{array}{cc} \rho_{22}{\left|u_{22}\right|}^{2} & \rho_{23}u_{22}^{*}\\ \rho_{32}u_{22} & 1-\rho_{22}{\left|u_{22}\right|}^{2} \end{array}\right),\\ {\hat{U}}_{2}^{†}{\hat{\sigma }}_{6}{\hat{U}}_{2}\Rightarrow {\hat{\rho }}_{5}^{\prime }=\left(\begin{array}{cc} \rho_{33}{\left|u_{21}\right|}^{2} & \rho_{32}u_{21}^{*}\\ \rho_{23}u_{21} & 1-\rho_{33}{\left|u_{21}\right|}^{2} \end{array}\right),\;{\hat{\rho }}_{6}^{\prime }=\left(\begin{array}{cc} 1-\rho_{33}{\left|u_{22}\right|}^{2} & \rho_{23}u_{22}\\ \rho_{32}u_{22}^{*} & \rho_{33}{\left|u_{22}\right|}^{2} \end{array}\right)\;. \end{array}$$

**Figure 1:**
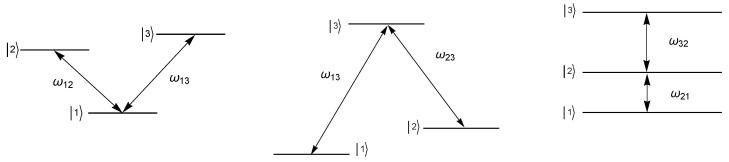
State configurations for the V- (**left**), the Λ- (**center**), and the Ξ-level (**right**) systems.

These transformations in many of the cases can represent the spontaneous-emission quantum channel. As in the other examples studied above, when the unitary matrices are rotated by angle θ=ωt, the original qubit systems can be recovered at times t=2πl/ω; l=0,1,2,…. It is important to mention that the states represented by Equation (15) correspond to three-level systems, where one of the levels is a dark state, and then only two of the levels can be populated, which have been experimentally obtained [24]. These kinds of systems have been of relevance as they can be created by two-photon processes in a three-level system [25] or by the adiabatic variation of the Rabi frequencies associated with the transitions between the three states [26]. For example, to obtain the state ${\hat{\sigma }}_{4}$, one can think of an atomic Λ-type three-level system (|1〉, |2〉, |3〉), which interacts with an environment [26]; see Figure 1. The Hamiltonian associated with this system can be written in the form:

$$\hat{H}=\left(\begin{array}{ccc} \omega_{1} & 0 & \omega_{13}\\ 0 & \omega_{2} & \omega_{23}\\ \omega_{13} & \omega_{23} & 0 \end{array}\right)\;,$$

where $\omega_{1,2}$ are the energies of the states |1〉,|2〉, respectively. By considering the energy of the ground state |3〉 equal to zero, $\omega_{13}$ and $\omega_{23}$ are the transition energies. Taking the zero energy in the ground state |3〉, we can make the replacements $\omega_{13}\to \omega_{1}\;e^{-i\omega_{1}t}$ and $\omega_{23}\to \omega_{2}\;e^{-i\omega_{2}t}$. The time evolution of the density matrix can be obtained by the expression:

(17) $$\frac{d}{dt}\hat{\rho }=i\left[\hat{\rho },\hat{H}\right]+{\hat{\rho }}^{\prime }\;,$$

where the matrix ${\hat{\rho }}^{\prime }$ is given by the interaction of the original density matrix with the environment:

$${\hat{\rho }}^{\prime }=\left(\begin{array}{ccc} \gamma_{31}\rho_{33} & -\gamma^{\prime }\rho_{12} & -\gamma_{1}\rho_{13}\\ -\gamma^{\prime }\rho_{21} & \gamma_{32}\rho_{33} & -\gamma_{2}\rho_{23}\\ -\gamma_{1}\rho_{31} & -\gamma_{2}\rho_{32} & -\gamma \rho_{33} \end{array}\right),$$

where the parameters $\gamma_{31}$, $\gamma_{32}$, and γ are the spontaneous-emission rates, which must satisfy $\gamma =\gamma_{31}+\gamma_{32}$, and the relaxation terms for the coherence components are named $\gamma_{1}$ and $\gamma_{2}$, which also satisfy $\gamma^{\prime }=\gamma_{1}+\gamma_{2}$. The resulting differential Equation (17) can be reduced by considering that the variation of the parameters $\rho_{13}$, $\rho_{23}$, and $\rho_{33}$ over time is smaller compared to the spontaneous emission and decoherence terms $\gamma_{31}$ and $\gamma_{32}$; this is called the adiabatic hypothesis. Under this hypothesis, it is possible to obtain a state with $\rho_{13}=\rho_{23}=\rho_{33}=0$, as the solution of the evolution of the density matrix ${\hat{\sigma }}_{4}$ discussed above.

Another way to obtain these types of systems is the case where the environmental interaction is neglected, i.e., ${\hat{\rho }}^{\prime }=0$ in Equation (17). The corresponding Schrödinger equation is $i\;\frac{d|\psi \rangle }{dt}=\hat{H}|\psi \rangle$, with $|\psi \rangle =a_{1}\left(t\right)e^{-i\omega_{1}t}|1\rangle +a_{2}\left(t\right)e^{-i\omega_{2}t}|2\rangle +a_{3}\left(t\right)|3\rangle$, which in view of the initial conditions $a_{1}\left(0\right)=\frac{\omega_{2}}{\sqrt{\omega_{1}^{2}+\omega_{2}^{2}}}$, $a_{2}\left(0\right)=-\frac{\omega_{1}}{\sqrt{\omega_{1}^{2}+\omega_{2}^{2}}}$, $a_{3}\left(0\right)=0$ leads to the solution:

$$a_{1}\left(t\right)=\frac{\omega_{2}}{\sqrt{\omega_{1}^{2}+\omega_{2}^{2}}}\;,\;a_{2}\left(t\right)=-\frac{\omega_{1}}{\sqrt{\omega_{1}^{2}+\omega_{2}^{2}}}\;;\;a_{3}\left(t\right)=0\;,$$

so the level |3〉 is never populated.

The density matrices ${\hat{\sigma }}_{5}$ and ${\hat{\sigma }}_{6}$ can be obtained by means of analogous procedures applied to the V and Ξ configurations of the three-level system depicted in Figure 1.

It is also important to mention that the unitary transformations defined by the matrices ${\hat{U}}_{1}$, ${\hat{U}}_{2}$, and ${\hat{U}}_{3}$ in Equation (5) can be generated experimentally by different proposed mechanisms, such as sliding mode control [27], adiabatic passage [28,29,30], and the robust control scheme [31,32]. We want to emphasize that the resulting quasi-unitary evolutions and the different quantum channels obtained in our work can have applications in quantum computing and quantum information theories. We think so because the quasi-unitary operations discussed here could be used as approximations to the standard quantum gates, and furthermore, the obtained quantum channels could also be used in the quantum correction algorithms found in the literature.

## 4. Probability Representation of the Qubit-State Evolution

In the quantum tomographic approach of qubit states [33,34], the states are identified with tomographic probability distributions. In the case of the minimal number of probability parameters, the density matrix of the qubit (spin-1/2) state reads [6]:

(18) $$\hat{\rho }=\left(\begin{array}{cc} p_{3} & p_{1}-1/2-i\left(p_{2}-1/2\right)\\ p_{1}-1/2+i\left(p_{2}-1/2\right) & 1-p_{3} \end{array}\right)\;,\;\sum_{j=1}^{3}{\left(p_{j}-\frac{1}{4}\right)}^{2}\leq \frac{1}{4}\;,$$

where $0\leq p_{k},\leq 1$ with k=1,2,3 are the probabilities to obtain the value +1/2 in the *x*, *y*, *z* axis, respectively. Thus, any qubit state can be identified through the probabilities $p_{1}$, $p_{2}$, and $p_{3}$, i.e., given the density operator, one can get the set $\hat{\rho }↔p_{1},p_{2},p_{3}$ and vice versa. In the case of qubits (1) associated with the qutrit state, the evolution of the probabilities after the unitary operation of the qutrit is determined by Equation (2). For example, we have a probabilistic representation corresponding to ${\hat{\rho }}_{5}^{\prime }$ in the first formula of Equation (17), i.e.,

(19) $$p_{3}\to p_{3}{\left|u_{11}\right|}^{2},\;p_{1}-1/2-i\left(p_{2}-1/2\right)\to \left(p_{1}-1/2-i\left(p_{2}-1/2\right)\right)u_{11}^{*}\;.$$

The change of probabilities can be characterized by the evolution of the Tsallis and Shannon entropies. For example, in (19), the unitary matrix parameter $u_{11}$ determines the evolution of the Shannon entropy related to a coin probability distribution $\left(p_{3},1-p_{3}\right)$ (assume that we have two nonideal classical coins I and II in such a game as coin flipping, coin tossing, or heads (up, ⊕) or tails (down, ⊖), which is the practice of throwing a coin in the air and checking which side is showing when it lands, in order to choose between two alternatives $P_{k}$ or $\left(1-P_{k}\right)$; k=1,2). This evolution is of the form:

$$S\left(\hat{U}\right)=-p_{3}|u_{11}{|}^{2}\ln\left(p_{3}{\left|u_{11}\right|}^{2}\right)-(1-p_{3}|u_{11}{|}^{2})\ln\left(1-p_{3}{\left|u_{11}\right|}^{2}\right)\;.$$

This entropy, as a function of the unitary evolution applied to the qutrit state, characterizes some aspects of the open dynamics of qubits. We point out that, as for $p_{3}$, there exist other classical entropic characteristics associated with the evolution of $p_{1}$ and $p_{2}$ given by Equation (19).

## 5. Concluding Remarks

A new mechanism to study the open system evolution of a noncomposite qudit system was established. As an example of the general procedure, we considered a qutrit system. Associated with the qutrit system, one can define different qubit density matrices, which evolve in an open-like way when a unitary transformation is enacted on the qutrit.

The application of the resulting transformations for the qubits within the qutrit was also discussed. The quasi-unitary transformations obtained here might be used as an approximation to quantum gates, whereas the quantum channels could be employed in quantum correction protocols.

Different types of quantum channels can be observed using the qubit decomposition of a qutrit system. In particular, the phase damping and the spontaneous-emission channels were obtained using a unitary transformation acting on specific qutrit density matrices. The phase damping channel was obtained when a unitary transformation of the density matrix with one off-diagonal term equal to zero was performed. A spontaneous-emission channel can be observed by unitary transformations acting over a dark state, i.e., a three-level state where one of the levels cannot be populated.

In addition to these channels, quasi-unitary transformations of the qubit states can be defined. This was also done by the application of a unitary matrix to the generic qutrit state.

The entropy evolution of the tomographic-probability distributions determined by the system of qubits was discussed.

We can extend our analysis to other qudit systems without subsystems since, o an arbitrary spin-*j* density matrix and the spin unitary evolution, one can associate the smaller spin $j^{\prime }<j$ evolution.

The possible experimental implementation of the procedure was also addressed, given that there exist several proposed ways to generate the unitary transformations such as by sliding mode control [27], adiabatic passage [28,29,30], or the robust control scheme [31,32].
